# Gene Expression Profiling and Prognostic Significance of Nuclear and Membrane Progesterone Receptors in Head and Neck Squamous Cell Carcinoma

**DOI:** 10.3390/ijms27041853

**Published:** 2026-02-14

**Authors:** Josipa Jelačić, Nina Milutin, Ilijana Stojković, Ozren Vugrinec, Ana Kvolik Pavić, Sanja Vušković, Ivan Mumlek, Vedran Zubčić, Dinko Leović, Mario Bilić, Petar Ozretić

**Affiliations:** 1 Laboratory for Hereditary Cancer, Division of Molecular Medicine, Ruđer Bošković Institute, 10000 Zagreb, Croatia; josipa.jelacic@irb.hr (J.J.); stojkovic.ilijana@gmail.com (I.S.); 2 Laboratory of Molecular Virology and Bacteriology, Division of Molecular Medicine, Ruđer Bošković Institute, 10000 Zagreb, Croatia; nmilutin@irb.hr; 3 Department of Otolaryngology, Head and Neck Surgery, University Hospital Centre Zagreb, 10000 Zagreb, Croatia; ozrenvugrinec@gmail.com (O.V.); dinko.leovic@gmail.com (D.L.); mbilic100@gmail.com (M.B.); 4 Department of Maxillofacial and Oral Surgery, University Hospital Osijek, 31000 Osijek, Croatia; kvolik-pavic.ana@kbco.hr (A.K.P.); zubcic.vedran@kbco.hr (V.Z.); 5 Faculty of Medicine Osijek, Josip Juraj Strossmayer University of Osijek, 31000 Osijek, Croatia; 6 Department of Oncology, University Hospital Centre Zagreb, 10000 Zagreb, Croatia; sanja.sandrk@gmail.com; 7 Bexorg, Inc., New Haven, CT 06419, USA; imumlek@gmail.com

**Keywords:** head and neck squamous cell carcinoma, sex hormone receptors, progesterone receptors, membrane progesterone receptors, biomarkers

## Abstract

The risk of developing some types of head and neck squamous cell carcinoma (HNSCC) is seven times higher in males, and such disparities may not be associated only with tobacco and alcohol consumption or HPV infection. Therefore, the endocrine microenvironment is considered another risk factor, as epidemiologic studies have unequivocally shown the protective effect of estrogen in women. This research was focused on progesterone receptors (PRs), the least-studied sex hormone receptors in HNSCC. Our study included fresh tissue samples from 95 primary tumors, 25 metastatic lymph nodes and 40 healthy oral mucosa. Gene expression of nuclear (*PGR*) and seven membrane PRs (*PAQR5*, *PAQR6*, *PAQR7*, *PAQR8*, *PAQR9*, *PGRMC1* and *PGRMC2*) was analyzed by qRT-PCR and associated with clinicopathological characteristics. The results showed that, compared to control tissue, *PGR* was increased in metastatic lymph nodes, while *PAQR5*, *PAQR7*, *PAQR8* and *PAQR9* were decreased in primary tumors (all *p* < 0.05). The expression of almost all PRs was greater in older patients and showed moderate to strong positive mutual correlations in both tumors and controls. *PARQ8* and *PAQR9* were increased in females and pT4 tumors (all *p* < 0.05). Survival analysis showed that higher *PAQR5* (hazard ratio (HR) 2.8, 95% confidence interval (CI) 1.19–6.57, *p* = 0.019) and *PAQR7* (HR 2.0, 95% CI 1.01–3.81, *p* = 0.048) were associated with worse overall survival, but their independence was not proven in multivariate analysis. Although most PRs were reduced in primary tumors, an increased *PAQR5* expression, also associated with tumor invasion markers, could likely mark a specific aggressive, advanced stage of primary tumors and potentially serve as a negative prognostic biomarker for HNSCC.

## 1. Introduction

Head and neck squamous cell carcinoma (HNSCC) represents a heterogeneous group of malignancies originating in the mucosal epithelium of the oral cavity, pharynx, and larynx [1]. As one of the most prevalent cancers globally, it accounts for approximately 5% of all cancer diagnoses and cancer-related deaths [2]. HNSCC is strongly associated with established risk factors such as tobacco use, alcohol consumption, and infection with high-risk human papillomaviruses (HPVs) [3]. Despite advancements in diagnostic and therapeutic approaches, the overall prognosis for HNSCC patients remains poor, particularly for advanced-stage disease [4]. The pathogenesis of HNSCC involves complex genetic, epigenetic, and microenvironmental alterations, underscoring the need for a deeper understanding of the molecular mechanisms driving its initiation and progression. Interestingly, sex differences in the pathogenesis of HNSCC have gathered increasing attention in recent years. Epidemiological studies consistently demonstrate that men have a two- to seven-fold higher risk of developing various types of HNSCC compared to women [5]. This disparity is often attributed to behavioral differences, including higher rates of tobacco and alcohol consumption in men [6]. However, intrinsic biological differences, such as hormonal influences and sex-specific gene expression, are increasingly recognized as significant contributors to the observed differences in HNSCC susceptibility and progression [7]. Recent research has focused on identifying novel biomarkers for early detection of HNSCC, prognostic assessment, and the development of targeted therapies. Due to the gender-specific risk for the development of HNSCC, part of the research is focused on the role of sex hormones and their receptors in the pathogenesis of this disease [5,8]. While estrogen and androgen receptors have been more extensively studied in HNSCC, the focus of our research was particularly on progesterone receptors (PRs), the least studied sex hormone receptors in this disease, which are classically associated with reproductive tissue but are also implicated in various cancers, including breast, ovarian and endometrial malignancies [9], with the large family of membrane progesterone receptors (mPRs) remaining especially unexplored in HNSCC. Taken together, these considerations provided the rationale for a comprehensive analysis of both nuclear and membrane PR gene expression in HNSCC.

Nuclear progesterone receptor (nPR), encoded by the *PGR* gene, is a ligand-activated transcription factor mediating the effects of progesterone (P4) by gene expression regulation. Since it is a member of the nuclear receptor superfamily, it influences many physiological processes, particularly those related to the menstrual cycle, pregnancy and embryogenesis [10]. Progesterone mediates its effects via both classical genomic pathways, involving transcriptional regulation and non-genomic signaling mechanisms that elicit rapid cellular responses [11]. The nPR exists as the isoforms PR-A (94 kDa) and PR-B (120 kDa), which, following ligand binding, form dimers and translocate to the nucleus to bind specific progesterone response elements (PREs) in target gene promoters [12]. This binding recruits co-regulators (co-activators or co-repressors) that modulate nPR-mediated gene expression, contributing to diverse physiological responses [11]. Computational studies have suggested that, beyond its classical role as a ligand-activated transcription factor, the progesterone receptor may also engage in direct protein–protein interactions, including a potential physical interaction with components of the Hedgehog-GLI signaling pathway, highlighting additional layers of regulatory complexity in nuclear receptor biology [13].

Membrane progesterone receptors, also known as non-classical PRs, are typically located on the cell membrane and can be categorized into the membrane progestin and adipoQ receptor (PAQR) family and the progesterone receptor membrane component family (PGRMC) [14,15]. The PAQRs are part of the G protein-coupled receptor (GPCR) superfamily, despite lacking structural and sequence homology with GPCRs and nuclear steroid receptors, and they include mPRα (*PAQR7*), mPRβ (*PAQR8*), mPRγ (*PAQR5*), mPRδ (*PAQR6*) and mPRε (*PAQR9*) [16]. In mammals, these receptors are differentially expressed in reproductive and non-reproductive tissues and exhibit high progestin binding affinity [17]. Among the PAQRs, mPRα is the most studied due to its widespread expression, while data on other members remain limited [18]. mPRs have been implicated in the development of hormone-sensitive cancers, including breast, ovarian, endometrial and prostate cancer [19]. The PGRMC family represents the second group of non-classical progesterone receptors, also called membrane-associated progesterone receptors (MAPRs), and includes PGRMC1, PGRMC2, neudesin (*NENF*) and neuferricin (*CYB5D2*). These receptors share a non-covalent heme-binding domain similar to cytochrome b5, which interacts with cytochrome P450 systems. PGRMC1 and PGRMC2 were first identified as hem-1 domain proteins [20], and PGRMC1 has been shown to physically interact with mPRα, suggesting potential crosstalk between the two receptor families [21].

Emerging evidence suggests that PRs may also play a role in the biology of HNSCC [22], although the exact mechanisms remain incompletely elucidated. PRs can influence tumor progression through their interactions with steroid hormones and downstream signaling pathways, which may vary between sexes influenced by differences in hormone levels [23]. Despite these insights, research on PRs remains in its infancy. Although accumulating evidence indicates the presence and potential importance of both nuclear and membrane PRs in HNSCC, their expression patterns and clinical implications remain incompletely defined and, in some cases, controversial. Therefore, in this study, we aimed to systematically analyze the gene expression of PRs in a large cohort of HNSCC and control tissue samples and to evaluate their associations with clinicopathological characteristics. To the best of our knowledge, this is the first comprehensive study that addresses the role of both nuclear and membrane PRs in HNSCC, and as our understanding of sex-specific biology in HNSCC deepens, integrating the role of PRs offers valuable perspectives on personalized treatment strategies.

## 2. Results

### 2.1. Clinicopathological Characteristics of the Study Cohort

This study included 160 samples (95 primary HNSCC samples, 25 metastatic lymph node samples and 40 control samples). The clinicopathological characteristics of the study cohort are summarized in Table 1. Patients with primary tumors were significantly older than the controls (median age 63 years, range 39–90, vs. 56.5 years, range 35–89; *p* < 0.0001). Both groups showed a male predominance but no significant difference in sex distribution. In contrast, smoking was significantly more frequent among patients than controls (71.0% vs. 25.0%, *p* < 0.0001). Most tumors originated in the oral cavity (55.8%), followed by the larynx (21.1%), oropharynx (8.4%), hypopharynx (7.4%), and nasal cavity or paranasal sinuses (7.4%). The majority of tumors were advanced at diagnosis, with pT3–pT4 categories accounting for 70.6% of cases, while 47.6% of patients had pathologically confirmed nodal metastases and 58.8% were classified as stage IV. Moderately differentiated carcinomas predominated (59.3%), whereas HPV positivity (14.7%) and ECS (23.5%) were observed in a minority of cases. LVI and PNI were present in 26.2% and 38.8% of tumors, respectively. At the end of a follow-up, 58.1% of patients were alive, whereas 41.9% had died.

### 2.2. Associations Between Clinicopathological Characteristics of Primary HNSCC Patients

As expected, the highest pathological stage was associated with the presence of tumor invasion markers, such as ESC (*p* = 0.0001; Supplemental Table S1) and LVI (*p* = 0.028), as well as HPV positivity (*p* = 0.041). Both invasion markers were also more frequently present among patients who died during the follow-up period (*p* = 0.003 and 0.007, respectively). The highest pathological grade was predominant in laryngeal carcinoma, while lower grades were most frequently observed in oral cancers (*p* = 0.013). In addition, G4 was, interestingly, less frequent among smokers than non-smokers (*p* = 0.021). On the other hand, smoking was expectedly associated with a higher nodal burden (*p* = 0.012) and predominated in males (*p* = 0.002) and among laryngeal and oral cancer patients (*p* = 0.003). A higher nodal burden and the presence of ESC also predominated in males (*p* = 0.015 and 0.0002, respectively). Patients with hypopharyngeal and laryngeal cancers were significantly older, while patients with oropharyngeal cancers were younger (*p* = 0.037). Patients who were older at diagnosis also showed a trend in being associated with a higher mortality (*p* = 0.055).

### 2.3. Associations Between PRs Gene Expression and Clinicopathological Characteristics in HNSCC and Healthy Control Tissue

**Figure 1:**
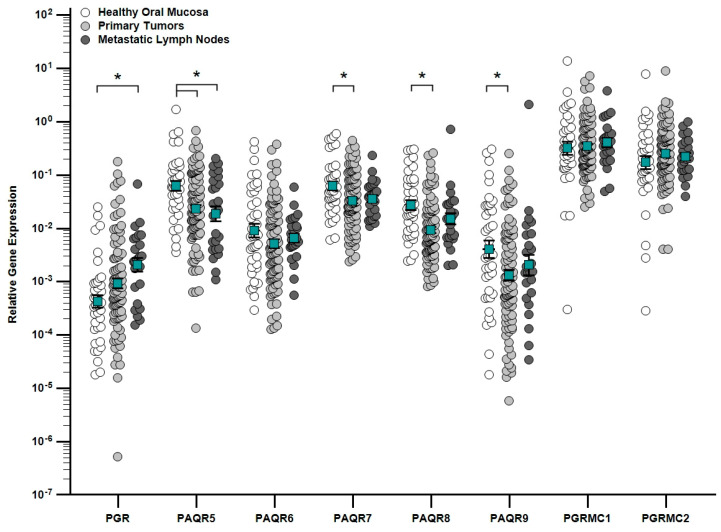
Relative gene expression of nuclear (*PGR*) and membrane (*PAQR5–9*, *PGRMC1–2*) progesterone receptors in healthy oral mucosa (white circles), primary tumors (light gray circles), and metastatic lymph nodes (dark gray circles). Each dot represents an individual sample; turquoise squares indicate mean values. Data are presented as mean (green squares) ± SEM on a logarithmic scale; * *p* < 0.05.

The expression of nuclear *PGR* and membrane progesterone receptors (*PAQR5*, *PAQR6*, *PAQR7*, *PAQR8*, *PAQR9*, *PGRMC1*, and *PGRMC2*) was quantified by qRT-PCR in 160 tumor and control samples. *PGR* was upregulated in metastatic lymph nodes compared to healthy oral mucosa (*p* = 0.005), whereas *PAQR5* was downregulated in both primary tumors and metastases (*p* = 0.001) and *PAQR7* (*p* = 0.015), *PAQR8* (*p* < 0.001) and *PAQR9* (*p* = 0.034) were decreased only in primary tumors versus controls (Figure 1).

Bioinformatic analysis showed that *PGR* expression was higher in normal tissue than in both primary (*p* < 0.001) and metastatic (*p* = 0.010) tumors, whereas *PAQR6* expression was higher in both primary (*p* < 0.001) and metastatic (*p* = 0.021) tumors than in normal tissue. Compared with normal tissue, *PAQR7* and *PGRMC1* were increased (*p* = 0.006 and <0.001, respectively), while *PAQR8* and *PAQR9* were decreased (*p* < 0.001 and 0.005, respectively) only in primary tumors (Supplemental Figure S1).

**Figure 2:**
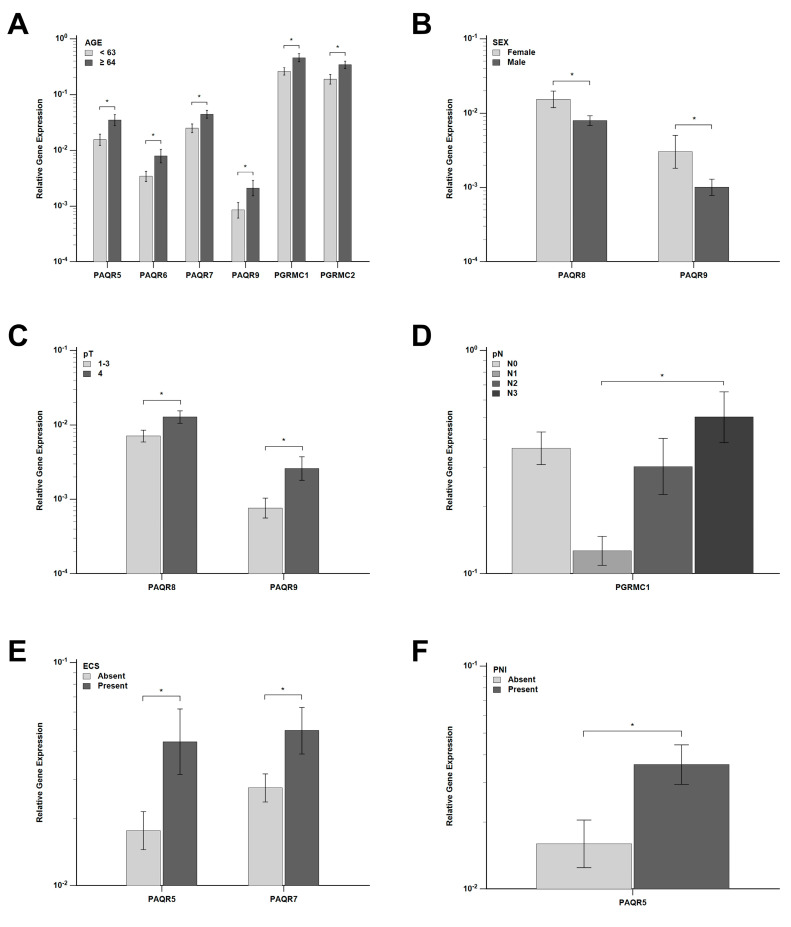
Relative gene expression of membrane progesterone receptors (*PAQR5-9*, *PGRMC1-2*) in primary tumor samples, stratified according to clinicopathological characteristics. (**A**) Higher expression of PAQR5, PAQR6, PAQR7, PAQR9, PGRMC1 and PGRMC2 in patients aged ≥ 64 years. (**B**) Increased PAQR8 and PAQR9 expression in female primary tumors. (**C**) Increased expression of same genes in pT4 compared to pT1–3 tumors. (**D**) Higher PGRMC1 expression in pN3 compared to pN1 tumors. (**E**) Increased PAQR5 and PAQR7 expression in ECS-positive tumors. (**F**) Increased PAQR5 expression in PNI-positive tumors. Data are presented as mean ± SEM on a logarithmic scale; * *p* < 0.05.

The gene expression of nPR and mPRs in primary tumors was evaluated in relation to clinicopathological characteristics. Participants were classified into two age groups according to the median age (63 years). Higher expression of *PAQR5*, *PAQR6*, *PAQR7*, *PAQR9*, *PGRMC1* and *PGRMC2* was observed in older patients (*p* = 0.014, 0.020, 0.017, 0.049, 0.014 and 0.019, respectively) (Figure 2A), while the same trend was also observed for *PGR* (*p* = 0.059) and *PAQR8* (*p* = 0.057). Significantly higher expression of *PAQR8* and *PAQR9* was detected in females compared to males (*p* = 0.029 and 0.037, respectively) (Figure 2B). With respect to primary tumor size, *PAQR8* and *PAQR9* expression were significantly increased in pT4 tumors compared to pT1–3 tumors (*p* = 0.034 and 0.013, respectively) (Figure 2C), with a similar trend for *PAQR7* (*p* = 0.083). Analysis of lymph node status revealed significantly higher *PGRMC1* expression in pN3 tumors compared to pN1 tumors (*p* = 0.042) (Figure 2D). In addition, tumors with ECS showed significantly higher expression of *PAQR5* and *PAQR7* compared to ECS-negative tumors (*p* = 0.025 and 0.047, respectively) (Figure 2E), whereas *PGRMC1* expression was also elevated but did not reach statistical significance (*p* = 0.085). Similarly, *PAQR5* expression was significantly increased in tumors with PNI (*p* = 0.021) (Figure 2F), with a similar trend observed for *PAQR6* and *PAQR8* (*p* = 0.057 and 0.058, respectively). No statistically significant differences in expression were observed for the remaining clinicopathological parameters (primary tumor site, smoking, HPV status, pathological stage and grade, LVI and survival status) (all *p* > 0.05). Supplementary Table S2 presents per-patient data on clinicopathological characteristics and PRs gene expression.

Because *PAQR9* expression is extremely low in the TCGA-HNSC dataset, its association with clinicopathological characteristics is unreliable. On the other hand, regarding age, only *PGRMC1* expression was statistically significantly higher in patients aged 41–60 years compared to the oldest patient group (aged 81–100 years) (*p* = 0.040). Regarding sex, *PAQR5* expression was higher in females (*p* = 0.014), whereas *PGRMC2* expression was higher in males (*p* = 0.032). The latter trend was also observed for *PAQR8* and *PGRMC1* (*p* = 0.065 and 0.062, respectively). The expression of *PGR* was highest in patients with N0 disease. On the contrary, *PAQR8* expression was highest in N2 patients, while *PGRMC2* expression was highest in N3. *PAQR5* expression was highest in stage 1 patients, while *PAQR7*, *PGRMC1* and *PGRMC2* expression was highest in stage 4 patients. *PGR* expression was highest in G2 and G3 patients, *PAQR5* in G1 patients, and *PAQR8*, *PGRMC1* and *PGRMC2* in G4 patients. Regarding the HPV status, *PAQR5* and *PAQR7* expression were higher in HPV-negative patients (*p* < 0.0001 and *p* = 0.026, respectively), while *PAQR8*, *PGRMC1* and *PGRMC2* were higher in HPV-positive patients (*p* < 0.0001, *p* = 0.007 and 0.0003, respectively) (Supplemental Table S3).

### 2.4. Correlation Between PR Gene Expression in Primary HNSCC and Healthy Control Tissue

**Figure 3:**
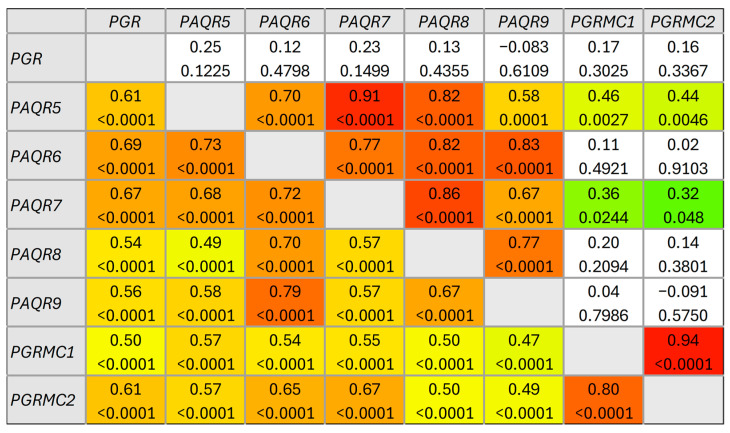
Correlations between PR gene expression in primary HNSCC tissue samples (N = 95) (lower triangle) and control tissue (N = 40) (upper triangle). For each gene pair, Spearman’s rank correlation coefficient (*ρ*) (upper value) and its *p*-value (lower value) are presented. Statistically significant positive *ρ*s are colored according to their extent.

A significant correlation was detected between the expression of the analyzed genes in both HNSCC and control samples. In primary HNSCC, the expression of all genes was mutually significantly correlated, and moderate positive correlation prevailed (*ρ* = 0.40–0.69). In contrast, strong positive correlation was observed between the expression of *PAQR5* and *PAQR6* and between *PGRMC1* and *PGRMC2* (Figure 3, lower triangle). Interestingly, in control tissue, the expression of fewer genes was mutually significantly correlated (Figure 3, upper triangle), while a strong positive correlation (*ρ* = 0.70–0.89) was observed among almost all PAQR genes. Here the correlation between *PGRMC1* and *PGRMC2* expression was very strong (*ρ* = 0.94, *p* < 0.0001), and there was no statistically significant correlation between *PGR* expression and the expression of any mPR gene.

### 2.5. Impact of Clinicopathological Characteristics and PR Expression Levels on Overall Survival of Croatian Patients with HNSCC

Furthermore, we wanted to assess the prognostic significance of PR expression in HNSCC patients. Survival data were collected for 86 (90.5%) patients with primary tumors, of whom 36 (41.9%) died during the follow-up period. The median follow-up time was 24 months (range 1–48 months). Overall survival (OS) was defined as the time from diagnosis to death from any cause or last check-up.

**Figure 4:**
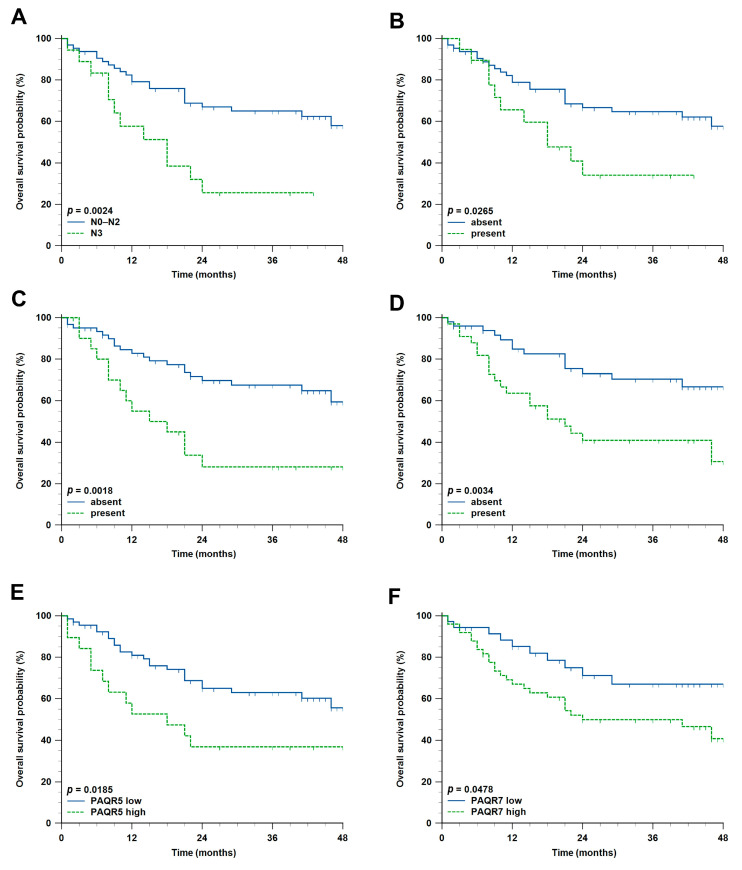
Kaplan–Meier survival curves of clinicopathological characteristics and PR expression levels associated with statistically significant shorter overall survival (OS) of Croatian patients with primary HNSCC (N = 86). (**A**) pN3 category; (**B**) presence of extracapsular spread (ECS); (**C**) presence of lymphovascular invasion (LVI); (**D**) presence of perineural invasion (PNI); (**E**) higher *PAQR5* expression; and (**F**) higher *PAQR7* expression. Tick marks represent censored cases.

The two-year OS rate was 58.4%. Statistically significant impact on OS was observed for several clinicopathological characteristics (Table 2). For example, pN3 patients had an approximately four times higher chance of dying compared with others (hazard ratio (HR) 4.2, 95% confidence interval (CI) 1.66–10.55, *p* = 0.002) (Figure 4A). Expectedly, all three tumor invasion markers—ECS, LVI and PNI—had a significant negative impact on HNSCC patient survival (Figure 4B–D). Regarding PR gene expression, higher *PAQR5* and *PAQR7* expression was also associated with shorter OS (HR 2.8, 95% CI 1.19–6.57, *p* = 0.019 and HR 2.0, 95% CI 1.01–3.81, *p* = 0.048, respectively) (Figure 4E,F). In addition, older age, pT4, pStage 4, and higher expression of *PAQR8* and *PAQR9* were associated with a negative impact on OS in primary HNSCC patients. Bioinformatic analysis showed that only higher *PGRMC1* expression showed a significant negative impact on OS (*p* = 0.0004) (Supplementary Figure S2g).

Finally, to determine whether the expression of any PR could be considered an independent prognostic biomarker for HNSCC, a Cox regression analysis was performed. Although *PAQR5* expression showed a significant impact on overall survival in univariate analysis, multivariate analysis showed that only pN and LVI were independent prognostic biomarkers in our cohort of Croatian HNSCC patients (Table 3).

## 3. Discussion

HNSCC remains one of the significant global health burdens, with persistently poor survival despite advances in diagnosis and treatment [4]. In recent years, growing recognition of sex disparities in HNSCC incidence and outcome has shifted attention toward the contribution of sex hormones and their receptors to HNSCC biology [7]. Within this framework, progesterone receptors, both classical nuclear and non-classical membrane-associated forms, have emerged as underexplored but potentially relevant modulators of tumor behavior, challenging the historical view of HNSCC as strictly hormone-independent. 

Against this background, an understanding of the clinicopathological characteristics of HNSCC patients remains essential for interpreting disease behavior and contextualizing potential biological contributors. In our study, the patient cohort was characterized by a median age comparable to that reported in the literature, consistent with epidemiological evidence indicating a higher incidence of head and neck cancers in older age groups [2,24]. Smoking was markedly more prevalent among patients than controls, confirming tobacco use as one of the major risk factors for head and neck carcinogenesis [25]. However, the observation that the highest histopathological grade (G4) was less frequent among smokers than non-smokers represents an unexpected finding, as smoking is generally associated with poorer differentiation and more aggressive tumor biology [26]. Regarding tumor localization, the oral cavity and larynx were the most affected sites, followed by the oropharynx and hypopharynx, which correspond to patterns reported in European and global populations [2]. Despite the limitation that samples were collected from only two Croatian hospitals, where maxillofacial surgeons at the University Hospital Osijek exclusively operate on oral cavity tumors, the observed distribution of tumors by anatomical site remains highly consistent with GLOBOCAN data. Likewise, the relatively low HPV positivity rate observed in our cohort may partly reflect the predominance of oral cavity tumors, which are less commonly HPV-associated [27] and may also be influenced by the implementation of a national HPV vaccination program in Croatia [28]. Although HPV positivity was relatively low overall, it showed a statistically significant association with higher pathological stage. This finding contrasts with the commonly reported association of HPV-positive tumors, particularly in the oropharynx, with earlier stage and more favorable prognosis [29]. However, this discrepancy may be explained by the predominance of non-oropharyngeal tumors in our cohort and the low absolute number of HPV-positive cases, which may limit direct comparability with HPV-enriched populations. On the other hand, in Croatia, as in other Eastern European countries, cancer patients are generally diagnosed at a more advanced stage [30]. Finally, the cohort showed a clear male predominance, and although associations between sex and aggressive pathological features remain inconsistent, male sex in our cohort was associated with a higher nodal burden and the presence of extracapsular spread. Nevertheless, despite the pronounced male predominance and sex-related clinicopathological differences observed in our cohort, HNSCC has historically been regarded as a hormonally independent malignancy.

Early studies of HNSCC conducted in the 1980s and 1990s generally concluded that these tumors do not express PRs and thus are considered hormonally independent, reporting very low or undetectable PR levels in tumor tissues [31,32]. However, subsequent studies yielded controversial and heterogeneous results, which may, at least in part, reflect the lack of standardized detection methods and the anatomical complexity of the head and neck region. Virolainen et al. reported nPR expression in 53% of laryngeal carcinoma samples, without an association with patient sex [33]. More recent studies have further challenged the concept of hormonal independence, demonstrating that nPR is expressed in a considerable proportion of HNSCC cases and is frequently co-expressed with estrogen receptors (ER) [22]. Specifically, nPR expression has been observed in approximately 49% of HNSCCs, with PR/ER co-expression detected in about 40% of cases [22]. In addition, nPR expression has been reported in 27% of oropharyngeal carcinomas, with lower expression observed in HPV- and p16-positive tumors [34]. Beyond nPRs, increasing attention has been directed toward *PGRMC1*. Several studies have shown that *PGRMC1* is significantly overexpressed in HNSCC tissues compared with normal mucosa and that its elevated expression is associated with unfavorable overall survival, suggesting its potential clinical relevance [35]. High *PGRMC1* expression has been consistently detected across anatomical subsites of HNSCC, with particularly strong immunohistochemical staining reported in tumors of the oral cavity and tongue [36]. Together, these findings from the literature highlight the heterogeneity of progesterone receptor expression in HNSCC and underscore the need for comprehensive analyses across different receptor subtypes and tissue compartments.

Relative gene expression analysis revealed significant differential expression of PRs, including *PGR* upregulation in metastatic lymph nodes and consistent downregulation of *PAQR5* across primary tumors and metastases. At the same time, *PAQR7*, *PAQR8* and *PAQR9* were downregulated exclusively in primary tumors. Partial concordance with the TCGA-HNSC analysis was observed for *PAQR5*, *PAQR8* and *PAQR9* in primary tumors In contrast, *PGR*, *PAQR6*, *PAQR7* and *PGRMC1* showed distinct expression patterns, likely reflecting differences in cohort composition and sample stratification between the two datasets. Notably, gene expression of several mPRs in primary tumors was associated with clinicopathological parameters. Higher expression of all mPRs, except *PAQR8*, in older patients suggests age-dependent modulation of progesterone receptor signaling. Sex-specific differences, most notably increased *PAQR8* and *PAQR9* expression in female tumors, further indicate potential hormonal influences on mPRs expression [37]. Moreover, increased expression of the same genes in larger primary tumors, together with elevated *PAQR5* and *PAQR7* expression in tumors with ECS and PNI, suggests an association between mPRs expression and locally aggressive tumor behavior. Interestingly, although *PGRMC1* was the most highly expressed PR in our cohort, no statistically significant differences were observed between control and tumor tissues. However, stratification by nodal status revealed significantly higher *PGRMC1* expression in pN3 tumors compared with pN1, indicating that *PGRMC1* expression may increase with advanced lymph node involvement. In contrast, TCGA-HNSC analysis revealed elevated *PGRMC1* expression in tumor samples compared with normal tissue, consistent with previous studies reporting increased *PGRMC1* expression in HNSCC and its association with poor overall survival [27]. Mechanistically, *PGRMC1* overexpression has been linked to metabolic reprogramming, including enhanced fatty acid metabolism, oxidative phosphorylation, and glutathione metabolism, which support tumor cell survival under hypoxic and nutrient-limited conditions [35]. Moreover, elevated *PGRMC1* expression has been associated with paclitaxel-tolerant persister cells and increased sensitivity to ferroptosis through PGRMC1-dependent lipophagy, highlighting its potential as a therapeutic target [38]. *PGRMC1* overexpression is frequently accompanied by gene copy number amplification and a higher prevalence of *PIK3CA* mutations, with PGRMC1-high tumors showing increased activation of pathways related to proliferation, invasion, and metastasis [35]. Consistently, strong PGRMC1 protein expression has been reported in oral cavity tumors, particularly of the tongue, further supporting its biological relevance in HNSCC [36].

In contrast to *PGR* and *PGRMC1*, the prognostic significance of other mPRs has not been investigated in the context of HNSCC. In our study, interestingly, all four members of the membrane progestin and adipoQ receptor (PAQR) family that showed statistically significantly decreased expression in primary tumors also showed either statistically significant (*PAQR5* and *PAQR7*) or borderline (*PAQR8* and *PAQR9*) association between higher expression and worse overall survival, of which *PAQR5*, whose increased expression was also associated with tumor invasion markers ECS and PNI and older age, could likely mark a specific aggressive, advanced stage of primary tumors and potentially serve as a negative prognostic biomarker for HNSCC. However, this definitely has to be confirmed in a much larger cohort of HNSCC patients, since studies associating increased *PAQR5* expression with worse survival are limited and confined mainly to in silico analyses in specific tumor contexts (e.g., hepatocellular carcinoma) [39]. In contrast, most clinically validated studies link higher *PAQR5* expression to favorable prognosis, like in endometrial [40] or kidney cancer [41].

In primary tumor tissue, the expression levels of all studied PR genes were mutually positively correlated, a pattern also observed in positive lymph nodes. In contrast, in healthy tissue there were no correlations between the expression of the PAQR family of genes and either the PGR or the PGRMC family of genes. Positive correlation between gene expression could potentially indicate a joint mechanism of expression regulation [42]. However, PRs genes are scattered across different chromosomes and no single “master regulator” has been identified to date. Furthermore, progesterone itself does not consistently induce the mPRs gene transcription [43]. However, it is known that estrogen can upregulate mPR expression, for example, *PAQR7* in reproductive tissues such as endometrium [44] or *PAQR8* in non-reproductive organs like brain [45], but this effect is usually indirect, i.e., does not happen through the classical estrogen response element (ERE), yet is rather mediated through the secondary transcription factors (AP-1, SP1, etc.) [46,47]. Both AP-1 and SP1 are frequently overexpressed and hyperactivated in HNSCC, where they drive proliferation, invasion, and inflammatory signaling [48,49]. Despite this, genes under SP1/AP-1 control may be selectively silenced in poorly differentiated tumors, thereby reducing PAQR gene expression and leading to a poor prognosis in HNSCC [50].

To sum up, although this study was conducted on a relatively large and well-characterized cohort, several limitations should be acknowledged. The samples were obtained from only two clinical centers in Croatia, which may have introduced center-specific and region-related bias and resulted in a predominance of oral squamous cell carcinoma within the cohort. In addition, the follow-up period was limited, thereby reducing the power of the survival analyses. Despite these limitations, our data provide a comprehensive transcript-level characterization of nPR and mPRs in HNSCC. Future studies should aim to validate these findings at the protein level, extend functional analyses using in vitro and in vivo models, and evaluate PR signaling in larger, multi-center cohorts with longer follow-up. Given the increasing complexity of PRs biology and the ongoing identification of novel progesterone-related signaling mechanisms, further investigation is warranted to fully elucidate their roles in HNSCC progression and therapeutic potential.

## 4. Materials and Methods

### 4.1. Tissue Samples and Patient Data Collection

A total of 120 fresh tumor tissue samples (95 primary HNSCCs and 25 metastatic lymph nodes) were collected between 2019 and 2023 at the Department of Otorhinolaryngology and Head and Neck Surgery, University Hospital Centre Zagreb and the Department of Maxillofacial and Oral Surgery, University Hospital Centre Osijek, while 40 healthy oral mucosa tissue samples that were used as a control were collected exclusively at the latter institution. Control tissue was intentionally collected from younger trauma patients and fewer smokers so that we could primarily collect oral mucosa tissue that was significantly shorter under the influence of both alcohol and tobacco. Clinical and pathological data were obtained from existing medical documentation, including the date of diagnosis, patient age, sex, smoking status, pathological TNM classification (pTNM), pathological tumor stage and grade, the presence of extracapsular spread (ECS), lymphovascular invasion (LVI) and perineural invasion (PNI), as well as survival information. This study was conducted according to the guidelines of the Declaration of Helsinki and approved by the Ethics Committees of the University Hospital Centre Zagreb (Class: 8.1-23/16-4, No.: 02/013 AG) and the Ethics Committee of the University Hospital Centre Osijek (No.: R2-1457/2022), and written informed consent was obtained from all participants.

### 4.2. HPV Detection by Polymerase Chain Reaction (PCR)

HPV was detected in HNSCC tissue as previously described [51]. Briefly, genomic DNA was extracted from freshly frozen tumor tissue samples through the phenol–chloroform method [52], and polymerase chain reaction (PCR) for HPV detection was performed with PGMY consensus primers. Type-specific primers for HPV 16, 18 and 33 were used in a multiplex PCR. The β-globin gene amplified with PC04/GH20 primers was used as an internal control for the quality of the isolated DNA, while the Ca Ski cell line (RRID: CVCL_1100) DNA was used as an HPV-positive control. The amplified products were visualized on an agarose gel using the Alliance 4.7 (UVITEC Cambridge, Cambridge, UK) imaging system.

### 4.3. Gene Expression Analysis by Quantitative Real-Time Polymerase Chain Reaction (qRT-PCR)

Total RNA was extracted from tissue samples using the Nucleozol reagent (Macherey-Nagel GmbH & Co. KG, Düren, Germany), and 1 μg of RNA was reverse transcribed using the High-Capacity cDNA Reverse Transcription Kit (Thermo Fisher Scientific, Waltham, MA, USA), all according to the manufacturers’ instructions. Gene expression was analyzed using the CFX Opus 96 Real-Time PCR System (Bio-Rad Laboratories, Hercules, CA, USA), with the SsoAdvanced Universal SYBR Green Supermix (Bio-Rad Laboratories, Hercules, CA, USA) and gene-specific primers. The qRT-PCR was carried out under the following conditions: initial denaturation at 95 °C for 30 s; 40 cycles of 95 °C for 10 s followed by 60 °C for 30 s; and finally melting curve analysis from 60 °C to 95 °C, with an increment of 0.5 °C. The results of relative gene expression were analyzed using the Bio-Rad CFX Maestro 1.0 Software v4.0. (Bio-Rad Laboratories, Hercules, CA, USA) and normalized to the housekeeping gene *RPLP0*, while the fold change was calculated using the 2^−ΔΔCt^ method [53]. Used primers are listed in Table 4, and primers covering both *PGR* isoforms (A and B) were used to determine the total PGR gene expression.

### 4.4. Statistical Analysis

The normality of the data distribution of continuous variables was assessed using the D’Agostino–Pearson test. Because all variables showed normal distributions after logarithmic transformation, an independent-samples *t*-test was used to infer differences between two groups and a one-way ANOVA with Tukey–Kramer post hoc test was used for more than two groups, all on log-transformed values. The correlation between PR gene expression levels was assessed by calculating Spearman’s correlation coefficient (*ρ*). Cut-off values used to dichotomize gene expression into ‘low’ and ‘high’ were determined by calculating the area under the receiver operator characteristic curve (AUC-ROC). The chi-squared test was used to assess associations between categorical variables. Survival curves were created with the Kaplan–Meier method, and a log-rank test was used for their comparison. The Cox proportional hazards regression with a stepwise variable selection was used for multivariate analysis. Statistical analysis was conducted using MedCalc Statistical Software version 23.4.2 (MedCalc Software Ltd., Ostend, Belgium). Two-tailed *p*-values less than 0.05 were considered statistically significant.

### 4.5. Bioinformatic Analysis

To externally validate our results, we utilized several on-line bioinformatic tools that integrate transcriptomic data from multiple sources, such as the Genomic Data Commons (GDC), Genotype-Tissue Expression (GTEx), and the NCBI Gene Expression Omnibus (GEO). For the comparison of gene expression of PRs in HNSCC primary tumors, metastases, and normal samples we used the TNMplot web tool (https://tnmplot.com/analysis/) (accessed on 15 December 2025) [56]. The relationship between PRs gene expression and clinical variables in HNSCC, such as tumor stage, patient gender and age, tumor grade, HPV status, and nodal involvement, was analyzed using the UALCAN platform (https://ualcan.path.uab.edu/) (accessed on 15 December 2025) [57]. The impact of PR expression on overall survival of HNSCC patients was analyzed using the OncoLnc tool (http://www.oncolnc.org/) (accessed on 15 December 2025) [58].

## 5. Conclusions

The mRNA expression levels of nuclear and all studied membrane PRs were detectable in HNSCC tissues of both primary tumors and positive lymph nodes, as well as in the healthy oral mucosa of unrelated, non-cancer patients. The highly expressed gene was mPR *PGRMC1*, and the nuclear *PGR* was lowly expressed. Although there were no significant differences in expression related to HNSCC site or HPV status, the majority of the studied mPR genes were downregulated in primary HNSCC compared with the healthy mucosa, whereas they were upregulated in older patients. In addition, increased expression of several mPRs was associated with worse OS of HNSCC patients, of which *PAQR5* could likely mark a specific aggressive, advanced stage of primary tumors and potentially serve as a negative prognostic biomarker for HNSCC.

## Figures and Tables

**Table 1:** Baseline clinicopathological characteristics of Croatian cohort of primary head and neck squamous cell carcinoma patients and healthy controls whose tissue samples were used in this study. Statistically significant *p*-values are in bold.

Characteristic	Patients	Controls	*p*-Value
	n (%)	n (%)	
Age (years) *	63 (39–90)	56.5 (35–89)	**<0.0001**
Sex			
Female	24 (25.3)	8 (20.0)	0.5130
Male	71 (74.7)	32 (80.0)	
Smoking			
No	27 (29.0)	30 (75.0)	**<0.0001**
Yes	66 (71.0)	10 (25.0)	
N.A.	2		
Site			
Nasal cavity and paranasal sinuses	7 (7.4)		
Oral cavity	53 (55.8)		
Oropharynx	8 (8.4)		
Larynx	20 (21.1)		
Hypopharynx	7 (7.4)		
Tumor size (pT)			
T1 (≤2 cm)	3 (3.5)		
T2 (>2 to ≤4 cm)	22 (25.9)		
T3 (>4 cm)	26 (30.6)		
T4 (invasion of adjacent/critical structures)	34 (40.0)		
N.A.	10		
Nodal involvement (pN)			
N0	44 (52.4)		
N1	7 (8.3)		
N2	14 (16.7)		
N3	19 (22.6)		
N.A.	11		
pStage			
1	1 (1.2)		
2	16 (18.8)		
3	18 (21.2)		
4	50 (58.8)		
N.A.	10		
Grade			
G1	3 (3.5)		
G2	11 (12.8)		
G3	51 (59.3)		
G4	21 (24.4)		
N.A.	9		
Human papillomavirus			
Negative	81 (85.3)		
Positive	14 (14.7)		
Extracapsular spread			
Absent	65 (76.5)		
Present	20 (23.5)		
N.A.	10		
Lymphovascular invasion			
Absent	62 (73.8)		
Present	22 (26.2)		
N.A.	11		
Perineural invasion			
Absent	52 (61.2)		
Present	33 (38.8)		
N.A.	10		
Survival			
Alive	50 (58.1)		
Deceased	36 (41.9)		
N.A.	9		

* Data are presented as median (range). N.A.—data not available; p—pathological.

**Table 2:** Impact of clinicopathological characteristics and PR expression levels on overall survival in a cohort of Croatian patients with primary HNSCC (N = 86). Presented are *p*-values for the log-rank test (statistically significant values are in bold) and hazard ratios and their 95% confidence intervals for characteristics with statistically significant log-rank test.

Characteristic	Categories	*p*-Values	HR (95% CI)
Age	≥64 vs. <63 years	0.0697	
Sex	female vs. male	0.6678	
Smoking	yes vs. no	0.9348	
HPV	positive vs. negative	0.6570	
pT	T1–T3 vs. T4	0.0579	
pN	N0–N2 vs. N3	**0.0024**	4.2 (1.66–10.55)
pStage	1–3 vs. 4	0.0545	
Grade	G1 vs. G2–G4	0.3538	
ECS	yes vs. no	**0.0265**	2.7 (1.12–6.68)
LVI	yes vs. no	**0.0018**	3.9 (1.66–9.14)
PNI	yes vs. no	**0.0034**	2.9 (1.42–5.92)
*PGR*	‘high’ vs. ‘low’	0.3252	
*PAQR5*	‘high’ vs. ‘low’	**0.0185**	2.8 (1.19–6.57)
*PAQR6*	‘high’ vs. ‘low’	0.1981	
*PAQR7*	‘high’ vs. ‘low’	**0.0478**	2.0 (1.01–3.81)
*PAQR8*	‘high’ vs. ‘low’	0.0578	
*PAQR9*	‘high’ vs. ‘low’	0.0704	
*PGRMC1*	‘high’ vs. ‘low’	0.4742	
*PGRMC2*	‘high’ vs. ‘low’	0.4947	

CI—confidence interval; ECS—extracapsular spread; G—tumor grade; HPV—human papillomavirus; HR—hazard ratio; LVI—lymphovascular invasion; N—lymph node involvement; p—pathological; PNI—perineural invasion; T—tumor size.

**Table 3:** Univariate and multivariate Cox proportional hazards regression analysis of overall survival in a cohort of Croatian patients with primary HNSCC (N = 86). Significant *p*-values are in bold.

Covariate	Categories	Univariate		Multivariate	
		HR (95% CI)	*p*-Value	HR (95% CI)	*p*-Value
Age	≥64 vs. <63 years	1.8 (0.94–3.58)	0.0769	-(-)	-
Sex	female vs. male	0.8 (0.38–1.85)	0.6712	-(-)	-
Smoking	yes vs. no	1.0 (0.48–2.20)	0.9357	-(-)	-
HPV	positive vs. negative	0.8 (0.31–2.08)	0.6608	-(-)	-
pT	T1–T3 vs. T4	1.9 (0.96–3.64)	0.0646	-(-)	-
pN	N0–N2 vs. N3	2.8 (1.40–5.79)	**0.0040**	2.9 (1.36–5.99)	**0.0057**
pStage	1–3 vs. 4	2.0 (0.97–4.05)	0.0617	-(-)	-
Grade	G1 vs. G2–G4	1.9 (0.46–8.06)	0.3676	-(-)	-
ECS	yes vs. no	2.2 (1.07–4.61)	**0.0322**	-(-)	-
LVI	yes vs. no	2.8 (1.42–5.67)	**0.0030**	3.00 (1.49–6.04)	**0.0021**
PNI	yes vs. no	2.7 (1.34–5.27)	**0.0053**	-(-)	-
*PGR*	‘high’ vs. ‘low’	1.4 (0.72–2.68)	0.3320	-(-)	-
*PAQR5*	‘high’ vs. ‘low’	2.2 (1.12–4.49)	**0.0231**	-(-)	-
*PAQR6*	‘high’ vs. ‘low’	1.7 (0.76–3.64)	0.2073	-(-)	-
*PAQR7*	‘high’ vs. ‘low’	2.0 (0.98–4.24)	0.0550	-(-)	-
*PAQR8*	‘high’ vs. ‘low’	1.9 (0.96–3.69)	0.0646	-(-)	-
*PAQR9*	‘high’ vs. ‘low’	1.8 (0.94–3.47)	0.0774	-(-)	-
*PGRMC1*	‘high’ vs. ‘low’	1.3 (0.65–2.49)	0.4796	-(-)	-
*PGRMC2*	‘high’ vs. ‘low’	1.5 (0.46–4.92)	0.5016	-(-)	-

CI—confidence interval; ECS—extracapsular spread; G—tumor grade; HPV—human papillomavirus; HR—hazard ratio; LVI—lymphovascular invasion; N—lymph node involvement; p—pathological; PNI—perineural invasion; T—tumor size.

**Table 4:** List of primer sequences used in RT-qPCR gene expression analysis.

Gene	Strand	Sequence (5′–3′)	Product Length (bp)	Reference
*RPLP0*	F	GGCACCATTGAAATCCTGAGTGATGTG	215	[54]
	R	TTGCGGACACCCTCCAGGAAGC		
*PGR*	F	AGGTCTACCCGCCCTATCTC	150	[55]
	R	TCCCACAGGTAAGGACACCA		
*PAQR5*	F	CAGCTGTTTCACGTGTGTGTGATCCTG	144	[55]
	R	GCACAGAAGTATGGCTCCAGCTATCTGAG		
*PAQR6*	F	CGCCTATCCATTCCTGTTCGAC	122	OriGene (HP215114)
	R	CGCAGAAGAGATGGTAGCCATG		
*PAQR7*	F	CAGCAGGTGGGTCCAGACATTCAC	122	[55]
	R	CGCTCTTCTGGAAGCCGTACATCTATG		
*PAQR8*	F	GTCCATCTGTACGCTCTCCC	106	[55]
	R	GCAGGCCATGTGGACAGATA		
*PAQR9*	F	GACGACTTCGTGGAGTGCTTCA	104	OriGene (HP219360)
	R	TTGAGCGTCTCGTTGGTAGGCT		
*PGRMC1*	F	TGACCTTTCTGACCTCACTGC	85	[55]
	R	GCCCACGTGATGATACTTGA		
*PGRMC2*	F	TCGAGAATGGGAAATGCAG	111	[55]
	R	TTGTGATCCTTGGTATCTTCTTCA		

F—forward; R—reverse.

## Data Availability

The original contributions presented in this study are included in the article/Supplementary Materials. Further inquiries can be directed to the corresponding author.

## Supplementary Materials

The following supporting information can be downloaded at https://www.mdpi.com/article/10.3390/ijms27041853/s1.
