# Supplementary material for: Gene Expression Profiling and Prognostic Significance of Nuclear and Membrane Progesterone Receptors in Head and Neck Squamous Cell Carcinoma
Source: Int J Mol Sci. 2026 Feb 14;27(4):1853. doi: 10.3390/ijms27041853 (PMC12940563; doi:10.3390/ijms27041853)
Supplement: Supplementary file 1 [file ijms-27-01853-s001.zip › ijms-4153585-Supplemental Figures.pdf]

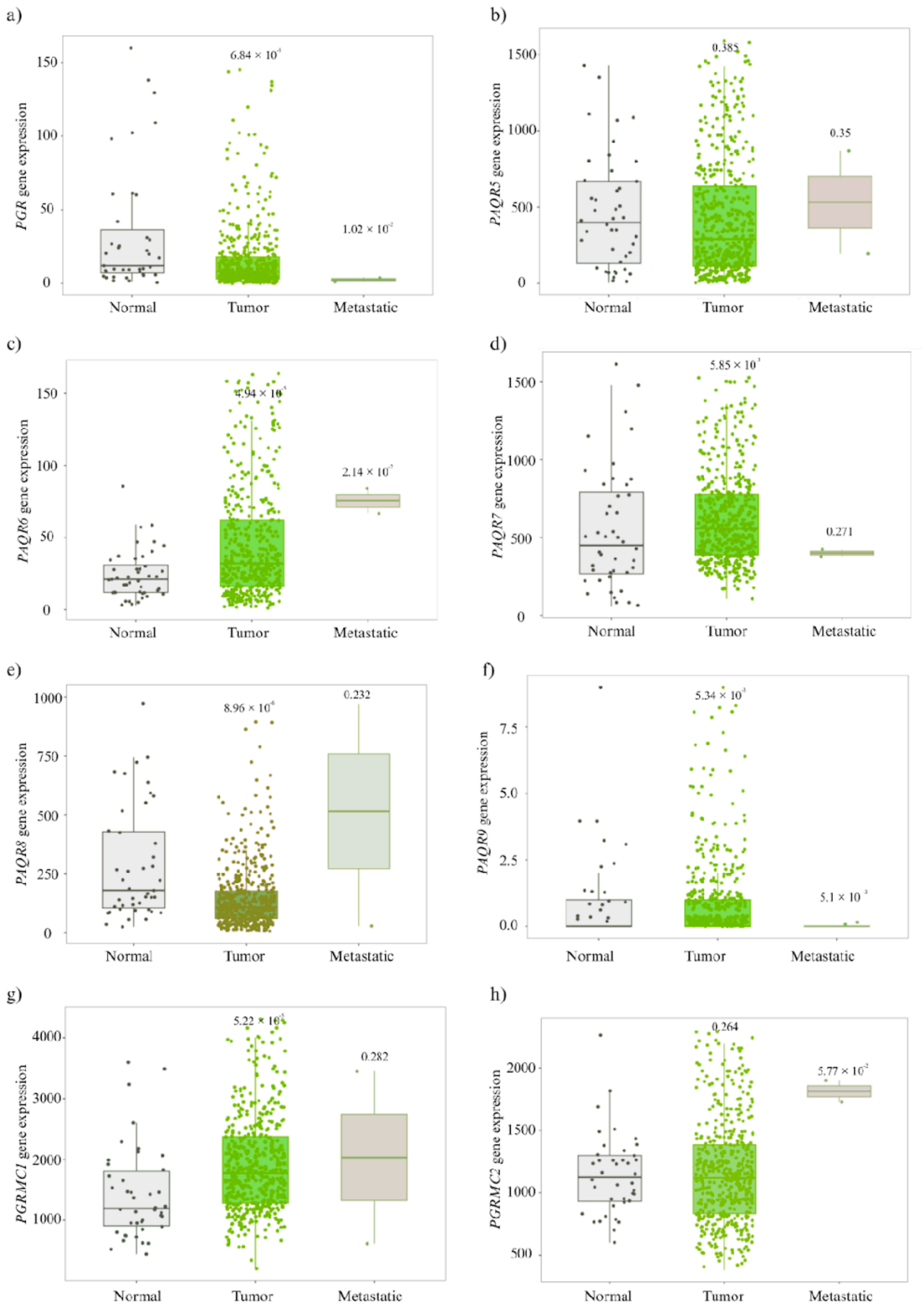

Supplemental Figure S1. Gene expressions of (**a**) *PGR*, (**b**) *PAQR5*, (**c**) *PAQR6*, (**d**) *PAQR7*, (**e**) *PAQR8*, (**f**) *PAQR9*, (**g**) *PGRMC1* and (**h**) *PGRMC2* in primary and metastatic HNSCC and normal healthy tissue samples from TCGA-HNSC dataset (TNMplot).

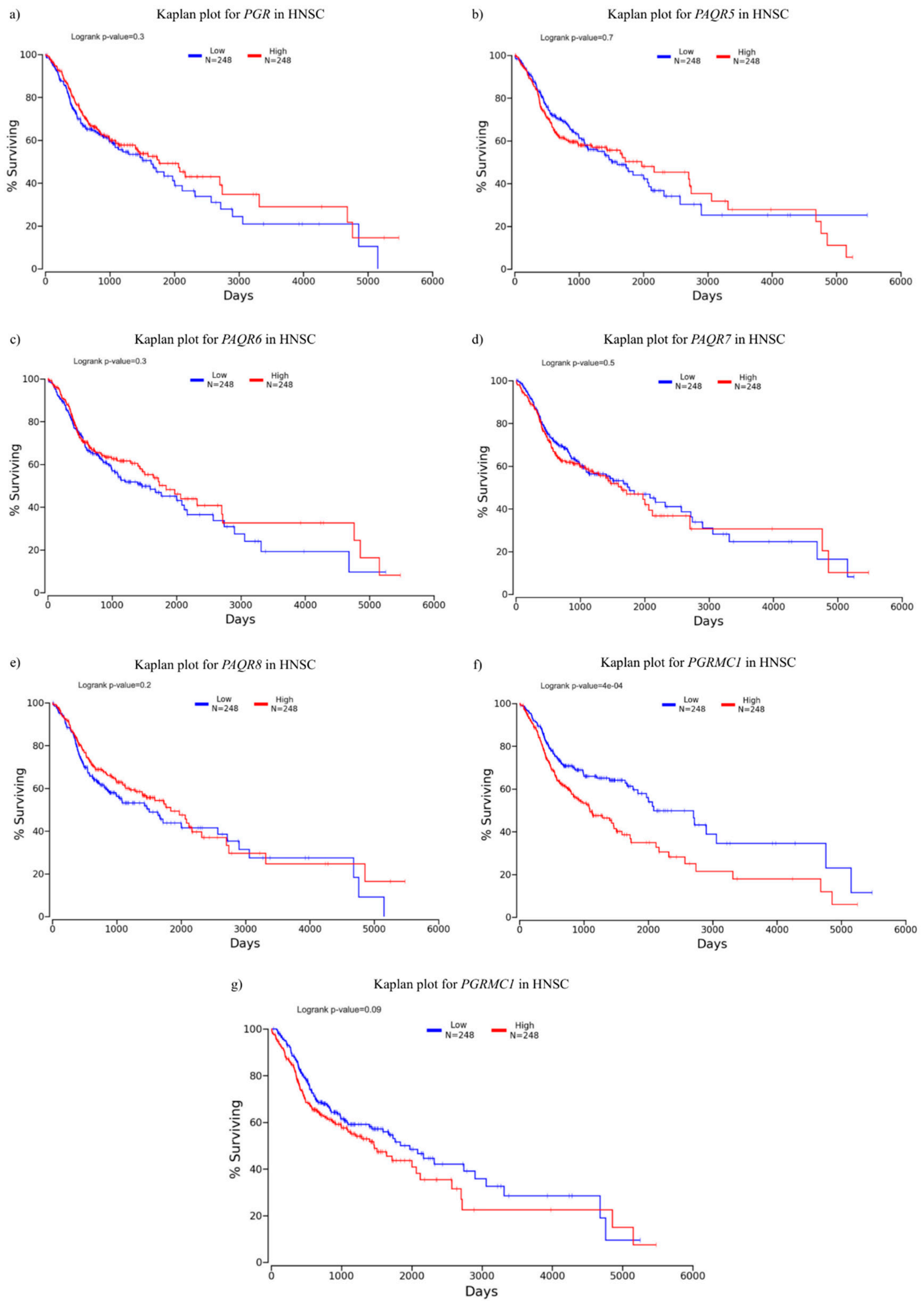

Supplemental Figure S2. Kaplan–Meier survival curves for overall survival of HNSCC patients from TCGA-HNSC dataset according to the levels of (**a**) *PGR*, (**b**) *PAQR5*, (**c**) *PAQR6*, (**d**) *PAQR7*, (**e**) *PAQR8*, (**f**) *PGRMC1* and (**g**) *PGRMC2* gene expression. Expression was binarized into 'low' and 'high' categories according to the median value. Presented *p*-values are for log-rang test (OncoLnc).
